# Early Diagnostic Markers and Risk Stratification in Sepsis: Prognostic Value of Neutrophil-to-Lymphocyte Ratio, Platelets, and the Carmeli Score

**DOI:** 10.3390/biomedicines13112658

**Published:** 2025-10-29

**Authors:** Mircea Stoian, Leonard Azamfirei, Andrei Claudiu Stîngaciu, Lorena-Maria Negulici, Anca Meda Văsieșiu, Andrei Manea, Adina Stoian

**Affiliations:** 1 Department of Anesthesia and Intensive Care, “George Emil Palade” University of Medicine, Pharmacy, Science, and Technology of Târgu Mureș, Gheorghe Marinescu Street No. 38, 540142 Târgu Mureș, Romania; mircea.stoian@umfst.ro (M.S.); leonard.azamfirei@umfst.ro (L.A.); 2 Intensive Care Unit, Mures Clinical County Hospital, Street Gheorghe Marinescu No 1, 540103 Târgu Mureș, Romania; andreistingaciu@yahoo.com; 3 Faculty of Medicine, “George Emil Palade” University of Medicine, Pharmacy, Science, and Technology of Târgu Mureș, Gheorghe Marinescu Street No. 38, 540142 Târgu Mureș, Romania; negulici.maria-lorena@stud19.umfst; 4 Department of Infectious Diseases, “George Emil Palade” University of Medicine, Pharmacy, Science, and Technology of Târgu Mureș, Gheorghe Marinescu Street No. 38, 540142 Târgu Mureș, Romania; anca-meda.vasiesiu@umfst.ro; 5 Department of Radiology, “George Emil Palade” University of Medicine, Pharmacy, Science, and Technology of Târgu Mureș, Gheorghe Marinescu Street No. 38, 540142 Târgu Mureș, Romania; 6 Doctoral School of Medicine and Pharmacy, “George Emil Palade” University of Medicine, Pharmacy, Science, and Technology of Târgu Mureș, Gheorghe Marinescu Street No. 38, 540142 Târgu Mureș, Romania; 7 Department of Pathophysiology, “George Emil Palade” University of Medicine, Pharmacy, Science, and Technology of Târgu Mureș, Gheorghe Marinescu Street No. 38, 540142 Târgu Mureș, Romania; adina.stoian@umfst.ro

**Keywords:** sepsis, critically ill patients, immunothrombosis, neutrophil-to-lymphocyte ratio, platelet dynamics, MDR risk, Carmeli score, risk stratification

## Abstract

**Background/Objectives:** Sepsis is characterized by a dysregulated host response to infection, where immune-inflammatory and thrombo-inflammation drive organ dysfunction. Early recognition of high-risk patients is essential. In addition, the increasing prevalence of multidrug-resistant (MDR) pathogens complicates therapeutic strategies, as delays in appropriate antimicrobial therapy are strongly associated with poor outcomes. **Methods:** We conducted a retrospective, single-center cohort study including 120 critically ill patients fulfilling Sepsis-3 criteria. Demographic, clinical, and laboratory data were collected at intensive care unit (ICU) admission, 48 h, and 72 h. The neutrophil-to-lymphocyte ratio (NLR) and platelet-to-lymphocyte ratio (PLR) were calculated from complete blood counts. At the same time, the Carmeli score was used as a surrogate for MDR infection risk. Prognostic accuracy was assessed using ROC curve analysis and multivariable logistic regression. **Results:** Persistently elevated NLR at 72 h and a delayed decline in platelet counts were associated with higher mortality. NLR at 72 h showed good predictive accuracy (AUC = 0.765; 95% CI 0.668–0.863), and the combination of APACHE II and NLR improved prognostic performance (AUC = 0.827). Importantly, the Carmeli score, reflecting MDR infection risk, was an independent predictor of outcome, linking antimicrobial resistance risk with sepsis prognosis. **Conclusions:** Dynamic immune-inflammatory biomarkers (NLR, platelets), when integrated with MDR risk assessment through the Carmeli score, provide a simple and cost-effective strategy for early prognostic stratification in sepsis. This combined approach may help facilitate early therapeutic decisions and patient care triage.

## 1. Introduction

Sepsis is a major global medical emergency, causing about 11 million deaths each year, nearly 20% of worldwide mortality [1]. Despite advances in antibiotic therapy, intensive care, and early detection, mortality among critically ill patients with sepsis remains high [2].

This pathological reaction involves inadequate activation of inflammatory pathways, leading to immunosuppression, disruption of the endothelial barrier, procoagulant activity, and tissue damage caused by hypoperfusion.

In the context of these pathophysiological disturbances, various hematological and biochemical changes occur early in the disease. For example, persistent leukocytosis indicates ongoing systemic inflammation and may reflect uncontrolled infection [3]. Conversely, lymphopenia occurs due to the apoptosis of T and B lymphocytes caused by proinflammatory cytokines such as TNF-α and IL-6. This condition is associated with a poor prognosis and secondary immunosuppression [4,5].

The neutrophil-to-lymphocyte ratio (NLR) combines these two aspects, acute inflammation and immune depletion, and has been identified as a predictive marker of severity and mortality in sepsis [6,7,8].

Thrombocytopenia is often seen in sepsis and can result from intravascular consumption, immune destruction, and changes in megakaryocyte production. Low platelet counts have been linked to more severe multiple organ dysfunction and a higher risk of death [9,10].

Haemoglobin and haematocrit levels may decrease due to haemodilution, blood loss, or erythropoietic dysfunction. Low albumin, an indirect indicator of capillary permeability and nutritional status, is also linked to a poor prognosis [11]. The increase in creatinine indicates impaired kidney function, which is one of the earliest and most common signs of organ dysfunction in sepsis [12]. Clinically, there is a need for simple, accessible, and relevant biomarkers to predict the course of critically ill patients. Standard laboratory tests, such as complete blood count, C-reactive protein (CRP), procalcitonin (PCT), creatinine, and albumin, can provide important clues about the severity of systemic inflammation, the risk of organ decompensation, and the progression to death [3,13,14]. Recent studies have shown that both isolated values and the dynamics of these parameters within the first 72 h after admission can provide valuable prognostic information regarding mortality [15].

Another relevant factor is the Carmeli score, initially validated for predicting infection with multidrug-resistant (MDR) organisms in hospitalized patients [16,17,18].

Although primarily used to guide empirical antibiotic therapy, it may also as an indirect marker of mortality risk by reflecting prior healthcare exposure, pathogen-related risk, and adequacy of empirical treatment. While many previous sepsis biomarker studies (including those on NLR) have been done in large multicentre cohorts, there remain few published data specifically from Eastern Europe, despite reports of high MDR pathogen prevalence in this region [19]. Validating these accessible markers in a high-resistance epidemiological setting may thus offer clinically valuable insights.

In addition to host dysregulation, the rising prevalence of MDR pathogens further complicates sepsis management. Delayed adequate antimicrobial therapy is strongly associated with poor outcomes. Therefore, integrating immune-inflammatory markers such as NLR and platelet count with MDR risk assessment (Carmeli score) could provide both prognostic and therapeutic insights.

This study aims to evaluate the prognostic value of hematological and inflammatory biological markers in patients with sepsis admitted to intensive care, focusing on changes within the first 72 h. Additionally, we examined the prognostic performance of the Carmeli score as an indirect indicator of MDR-related risk. The goal was not to find new predictors but to assess whether combining dynamic laboratory markers with clinical severity scores and Carmeli risk stratification provides additional prognostic information for in-hospital mortality.

## 2. Materials and Methods

### 2.1. Study Design and Investigated Population

This was a retrospective, single-center cohort study conducted at the Intensive Care Unit of Mureș County Clinical Hospital in Târgu Mureș, Romania. The retrospective design inherently limited data completeness and the ability to control for therapeutic interventions; this was acknowledged as a methodological limitation in the Discussion section. It included consecutive adult patients (≥18 years) admitted from 1 January 2023, to 31 December 2023, with a diagnosis of sepsis based on Sepsis-3 criteria [2], who were screened for eligibility. Patients were excluded if they presented with severe immunosuppression at admission (active chemotherapy, neutropenia < 1.0 × 10^9^/L, or known HIV infection), or major coagulopathies (platelet count < 20 × 10^9^/L). Cases with incomplete laboratory data at 0, 48, or 72 h were also excluded to ensure accuracy consistency.

A total of 120 patients met the inclusion criteria, and with complete laboratory data at admission, 48 h, and 72 h were included in the final analysis.

The study protocol was reviewed and approved by the Ethics Committee of Mures County Clinical Hospital (approval no. 20292, issued on 23 January 2025). The requirement for informed consent was waived because of the retrospective nature of the study.

### 2.2. Data Collection

Demographic data (age, sex) and clinical data (length of stay, SOFA, APACHE II, and Carmeli scores), as well as discharge status (survivor or non-survivor), were obtained from electronic medical records. The Carmeli score was applied as a surrogate indicator of MDR risk, as complete microbiological MDR phenotyping was unavailable for all cases. This methodological limitation was further discussed in the Limitations section. Laboratory values were assessed at three time points: admission (T0), 48 h (T1), and 72 h (T2). The following parameters were recorded: haematological markers (leukocytes, neutrophils, lymphocytes, platelets, haemoglobin, haematocrit); inflammatory markers (CRP, PCT); and biochemical parameters (albumin, creatinine, INR). For each time point, the NLR was calculated as the absolute neutrophil count divided by the absolute lymphocyte count. The platelet-to-lymphocyte ratio (PLR) was calculated as the absolute platelet count divided by the absolute lymphocyte count. Both indices were considered surrogate markers of systemic inflammation.

### 2.3. Microbiological Analysis

Microbiological data were documented at 72 h after intensive care unit (ICU) admission, including the presence or absence of identified pathogens and their broad categories (Gram-negative, Gram-positive, mixed infections, fungi). Antimicrobial susceptibility testing and MDR phenotyping were not conducted. Instead, the risk of infection with MDR was indirectly evaluated using the Carmeli score, which categorizes patients based on prior healthcare exposure and the likelihood of resistant pathogens. This score stratifies patients into three categories based on previous healthcare exposure (such as hospitalization, dialysis, or nursing home residence) and prior antibiotic use, with higher categories indicating increased MDR risk.

### 2.4. Statistical Analysis

Descriptive statistics were used to characterize the overall cohort. The Shapiro–Wilk test was used to assess the normality of continuous variables, helping determine the appropriate parametric or non-parametric statistical tests. Variables with a Gaussian distribution are presented as mean ± standard deviation (SD), while those with a non-Gaussian distribution are shown as median (interquartile range). Comparisons between survivors and non-survivors were performed using Student’s *t*-test, Mann–Whitney U test, ANOVA, Kruskal–Wallis, or Friedman test (depending on the distribution and number of groups compared). For ANOVA and Kruskal–Wallis tests, post hoc pairwise Wilcoxon signed-rank tests were applied for continuous variables. For categorical variables, the Chi-square test or Fisher’s exact test was used. Binary associations were quantified by calculating odds ratios (OR) with 95% confidence intervals. A *p*-value < 0.05 was considered statistically significant. Analyses were conducted using IBM SPSS Statistics, version 22 (IBM Corp., Armonk, NY, USA). The power analysis for the logistic regressions was conducted using G*Power software (Release 3.1.9.6) (Heinrich-Heine-Universität Düsseldorf) [20,21]. Multivariable logistic regression models were validated through bootstrapping with 1000 resamples. This nonparametric approach involved repeatedly sampling with replacement from the original dataset to evaluate the stability and precision of model coefficients, including confidence intervals and standard errors. Bootstrapping helped assess the robustness of the effect estimates, considering sample size and variability.

## 3. Results

### 3.1. Study Population

The cohort included 120 patients with sepsis according to Sepsis-3 criteria, of whom 50 (41.7%) survived and 70 (58.3%) died. The median age was 70.5 years (IQR 62–81), with 52.5% male. Age and sex did not differ significantly between survivors and non-survivors. SOFA scores were significantly higher in non-survivors (14.61 ± 3.34) compared to survivors (11.00 ± 3.71; *p* < 0.001), as were APACHE II scores (30.5 [25.75–38] vs. 22.0 [2.18–27]; *p* < 0.001). Although Carmeli scores had similar medians across groups, deceased patients showed significantly higher ranks (*p* = 0.001, Mann–Whitney U test). ICU length of stay was similar between groups. The distribution of significant comorbidities—including cardiovascular, pulmonary, renal, neurological, digestive, oncologic, and diabetic conditions—was broadly comparable, with no statistically significant differences (all *p* > 0.05). (Table 1).

### 3.2. Baseline Laboratory Parameters at ICU Admission (T0)

At admission, white blood cell count, neutrophils, lymphocytes, platelets, haemoglobin, albumin, and creatinine showed no significant differences between groups. NLR tended to be higher in non-survivors (17.19 [7.55–31.41]) compared to survivors (10.71 [7.04–20.96]), but this difference did not reach statistical significance at T0 (*p* = 0.076) (Table 2).

### 3.3. Early 72-h Trajectories of Inflammatory and Hematologic Markers

Temporal changes over 0–48–72 h were evaluated using non-parametric tests (Friedman for repeated measures and Wilcoxon for post hoc comparisons), given the non-parametric distribution of trajectories.

#### 3.3.1. Neutrophil-to-Lymphocyte Ratio (NLR)

At admission (T0), NLR was slightly higher in non-survivors than in survivors, but the difference did not reach statistical significance (Table 2). During the first 72 h, the trajectories diverged: survivors showed a continuous decline with a significant overall time effect (Friedman *p* < 0.001) and a sharp decrease between 48 h and 72 h (Wilcoxon post hoc *p* < 0.001). Conversely, non-survivors maintained persistently high NLR values with no significant change over the same period (*p* = 0.929). By 72 h, the median NLR in survivors was notably lower than in non-survivors, indicating a divergent inflammatory course. Although the discriminative ability of NLR was moderate overall, its very high specificity at 72 h suggests it could be especially useful for identifying high-risk patients rather than serving as a universal prognostic marker.

#### 3.3.2. Platelet Count (PLT)

At admission, platelet counts were similar between groups (Table 2). During the first 72 h, survivors maintained relatively stable platelet levels (Friedman *p* = 0.572). In contrast, non-survivors showed a late decline: values remained stable through 48 h, followed by a significant drop between 48 and 72 h (Wilcoxon post hoc *p* < 0.001), which was reflected in a significant overall time effect (*p* = 0.001). This different course supports PLT dynamics as a marker of unfavourable outcome. Details of medians [IQR] and post hoc comparisons are provided in Table 3.

### 3.4. Predictive Performance of Biomarkers at 72 h

**Figure 1:**
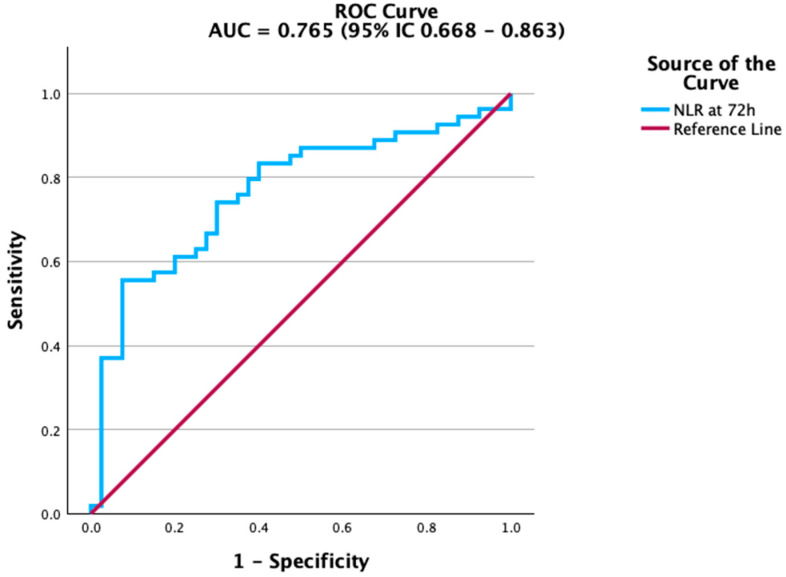
ROC curve of Neutrophil-to-Lymphocyte Ratio (NLR) at 72 h for predicting mortality. NLR demonstrated the strongest discriminative ability among the tested biomarkers.

To assess prognostic value, we conducted ROC analyses for biomarkers measured at 72 h (Table 4). Among all variables, NLR (AUC = 0.765, 95% IC 0.668–0.863) demonstrated the strongest ability to distinguish survivors from non-survivors (Table 4; Figure 1).

The NLR showed the best predictive performance, clearly distinguishing patients at risk of death from those who survived. As shown in Figure 1, the ROC curve for NLR had the steepest slope, indicating strong discriminatory ability.

**Figure 2:**
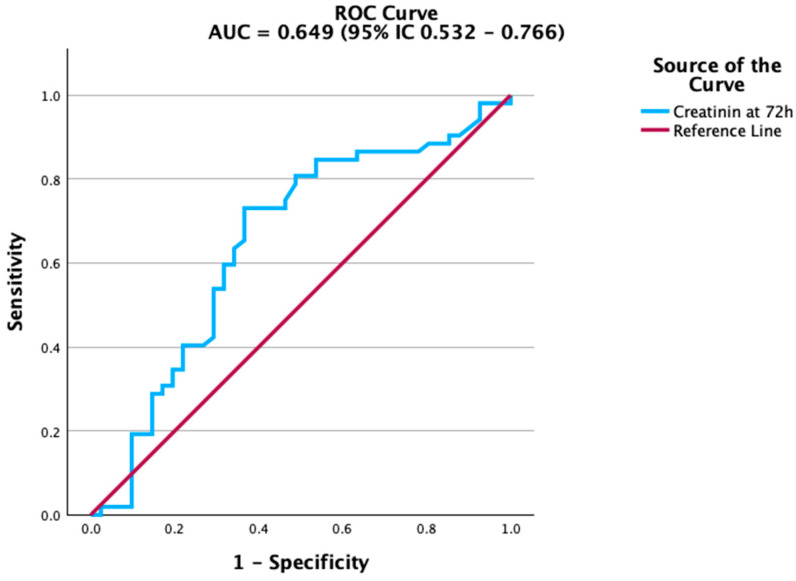
ROC curve of creatinine at 72 h for predicting mortality. Creatinine demonstrated modest but significant predictive ability.

**Figure 3:**
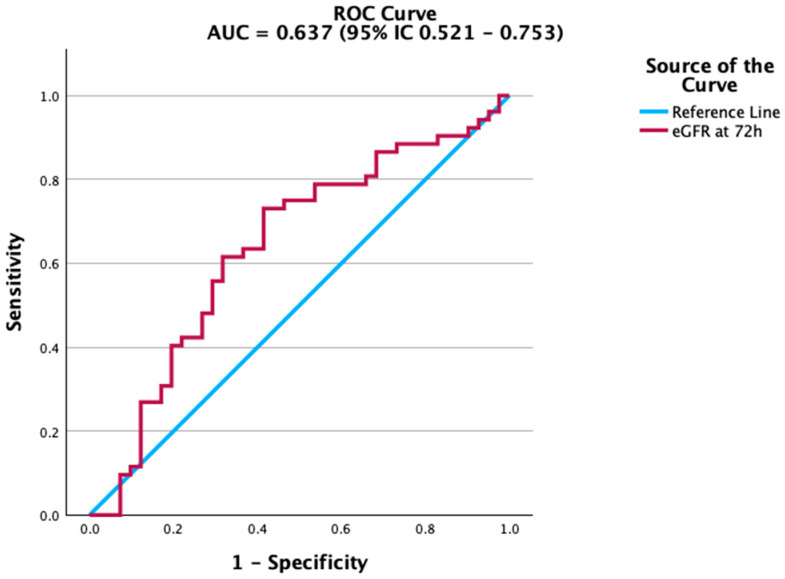
ROC curve of estimated glomerular filtration rate (eGFR) at 72 h for predicting mortality. eGFR shows moderate discriminatory ability.

Renal function markers also contributed to mortality prediction. Creatinine levels at 72 h were significantly associated with adverse outcomes, although the accuracy was modest. The ROC curve in Figure 2 highlights this association, while estimated glomerular filtration rate (eGFR) (Figure 3) showed a similar, albeit weaker, predictive capacity. In contrast, platelet count and albumin did not achieve statistical significance. Their ROC curves are therefore provided in the Supplementary Material (Figures S1 and S2) for transparency. This aligns with previous studies indicating that the prognostic value of platelet counts and albumin is more evident in trajectory-based or composite indices rather than as isolated parameters.

### 3.5. Association of Clinical Severity Scores with Mortality

Univariate logistic regression identified the Carmeli score as a significant predictor of mortality, while APACHE II showed only a borderline association, and SOFA was not significant (Table 5). When all three scores were included in a multivariable model, only the Carmeli score remained an independent predictor.

**Figure 4:**
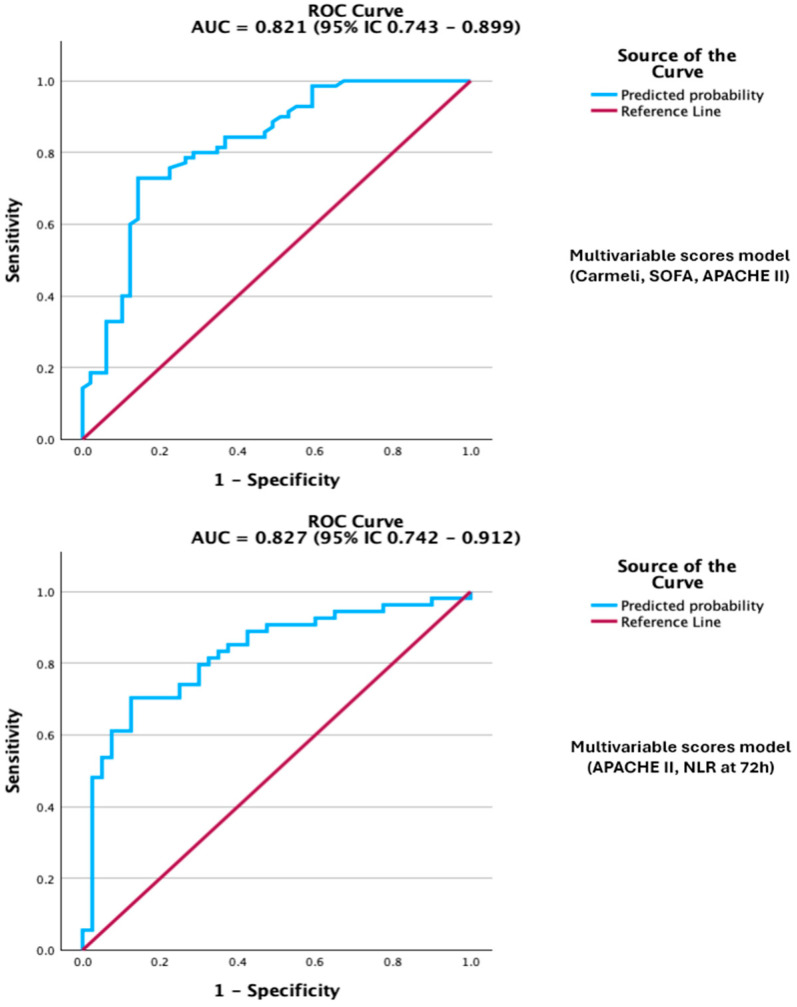
Legend: ROC curve of the multivariable scores model, Carmeli, SOFA, APACHE II, and model APACHE II + NLR at 72 h for predicting mortality. The models demonstrate excellent discrimination.

This probably reflects its role as an indirect indicator of multidrug resistance risk and the possible delay in starting effective empirical therapy, both well-known factors that influence sepsis mortality. ROC analysis showed good discriminative ability, with acceptable calibration and overall model fit. The Hosmer–Lemeshow goodness-of-fit test showed a good model fit (*p* = 0.066). Multicollinearity was also assessed with variance inflation factors (VIF) ranging from 1.05 to 3.32, all below the accepted threshold of 5. (Figure 4; see Supplementary Table S1 for complete diagnostics, including the Omnibus test, pseudo-R^2^ indices, Hosmer–Lemeshow, and classification metrics). Taken together, these findings emphasize that the Carmeli score is not only a prognostic marker but also a clinically relevant surrogate of MDR infection risk, reinforcing the therapeutic importance of timely initiation of adequate antimicrobial therapy in sepsis.

In this case, the standard events-per-variable (EPV) rule had a value of 23 (three predictors and 70 events), exceeding the recommended minimum of 10, which supports robust and stable model estimates. The power calculation for the sample size was 99.9% for the OR of 3.68 of the Carmeli score, confirming an adequate sample size to detect relevant effects. Internal validation of the model was performed using bootstrap resampling with 1000 samples, which showed stable coefficient estimates with bias-corrected and accelerated (BCa) 95% confidence intervals. This further supports the significant association of the Carmeli score with mortality (BCa 95% CI: 1.6 to 11.15). SOFA and APACHE II scores included an OR of 1, indicating nonsignificant effects and suggesting uncertainty in the predictor’s association with the outcome. After adjusting the model for age, sex, and comorbidities, none of these variables showed statistically significant associations (*p*-values > 0.05), indicating they did not independently contribute to predicting the outcome in this dataset. The model is included in the Supplementary Material as Table S2.

Univariate models were fitted separately for each score. Full model diagnostics (Omnibus test, pseudo-R^2^, Hosmer–Lemeshow, classification metrics) are reported in Supplementary Table S1. Abbreviations: OR, odds ratio; CI, confidence interval.

When all three scores were entered into a multivariable model, Carmeli remained the only independent predictor of death, underscoring its dominant role in risk stratification within this cohort. The discrimination of the multivariable model was very good on ROC analysis, while calibration and overall model fit were acceptable, with an AUC of 0.821. (Figure 4; Supplementary Table S1 for full diagnostics, including Omnibus, R^2^ indices, Hosmer–Lemeshow, and classification metrics).

### 3.6. Combined Model: APACHE II and NLR at 72 h

Beyond analyzing individual severity scores, we also explored supplementary models that combined clinical scores with inflammatory biomarkers. The combination of APACHE II with NLR at 72 h yielded the best prognostic performance, with an AUC of 0.827 (95% CI 0.742–0.912; *p* < 0.001) (Supplementary Table S3), outperforming each variable alone. However, this combined model should be considered exploratory, as it was developed from a relatively small, single-centre cohort and has not yet undergone external validation. In this two-variable model, both predictors remained independently associated with mortality, indicating they provide complementary prognostic information (Table 6). The combined model showed improved discrimination and good calibration compared with single-predictor models. Multicollinearity was evaluated using VIF, and the values were under 5 (1.125 for APACHE II score and 1.126 for NLR at 72 h). The ROC curve illustrated overall performance, while comprehensive diagnostics, including goodness-of-fit (*p* = 0.818), pseudo-R^2^ indices, classification metrics, and the Youden-derived optimal probability threshold, are available in the Supplementary Material (Supplementary Table S3).

Regarding internal validation, we used bootstrapping with 1000 resamples to evaluate model stability. The BCa 95% confidence intervals for APACHE II (1.02 to 1.16) confirmed its strong association with mortality. The BCa interval for NLR at 72 h (0.049 to 1.2) included OR = 1, indicating it was not statistically significant and warranting cautious interpretation of its predictive value. The EPV was approximately 35, surpassing the recommended minimum of 10, but the statistical power was low—14.15% for the APACHE II score and 8.5% for NLR at 72 h—due to minimal effect size in the studied population. After adjusting the model for age, sex, and comorbidities, none of these variables showed statistically significant associations (*p*-values > 0.05), suggesting they did not independently predict the outcome in this dataset. The model is provided in the Supplementary Material as Table S4.

This combined model was evaluated separately from the multivariate regression, which included all severity scores, and should therefore be considered a complementary approach rather than an alternative to the Carmeli score. Complete diagnostics, including ROC AUC, calibration indices, classification performance, and the Youden-derived optimal threshold, are provided in Supplementary Table S3.

### 3.7. Microorganisms Frequently Isolated

Within the study group (*n* = 120 patients with sepsis according to Sepsis-3), 31 patients (25.8%) had no identified pathogens at 72 h after ICU admission. In the remaining patients, Gram-negative and Gram-positive bacteria were isolated, as well as fungi (especially *Candida* spp.). The distribution of germ types was as follows: Gram-negative: 57 patients (47.5%); Gram-positive: 17 patients (14.2%); Gram-positive + Gram-negative: 15 patients (12.5%) (Supplementary Table S5).

In the detailed analysis of pathogens (Supplementary Table S5), a predominance of Gram-negative bacilli was observed, with *E. coli* (13.3%), *Klebsiella pneumoniae* (13.3%), *Acinetobacter baumannii* (10.8%), and *Pseudomonas aeruginosa* (9.2%) being the most common. Among the Gram-positive bacteria, methicillin-susceptible *Staphylococcus aureus* (MSSA, 16.7%) and *Enterococcus* spp. (8.3%) were identified. Less frequent isolates included MRSA (3.3%) and *Streptococcus pneumoniae* (4.2%). Notably, fungi, mainly *Candida* spp., were isolated in 26.7% of patients, which is a particularly relevant finding. This subgroup exhibited higher mortality; however, the statistical analysis did not reveal significant differences compared to patients without *Candida* (*p* = 0.108) (Supplementary Table S6). Systematic antimicrobial susceptibility testing and MDR phenotyping were not available for all isolates, which limited pathogen-specific resistance profiling.

## 4. Discussion

In our cohort of 120 critically ill patients meeting Sepsis-3 criteria, in-hospital mortality was 58.3%, which aligns with the highest reported global estimates. ICU mortality in septic shock varies between 355 and 60% depending on region and resources [22,23,24] reflecting differences in case-mix and antimicrobial resistance burden. These findings highlight the variability of sepsis outcomes across the world and emphasize regional differences in case-mix, antimicrobial resistance, and resource allocation.

As expected, traditional severity scores (SOFA and APACHE II) were significantly higher in non-survivors. These scores have long been validated as mortality predictors in sepsis and remain widely used in both research and clinical practice [2,25,26]. However, in our multivariable model, only the Carmeli score emerged as an independent predictor of mortality. Our findings indicate that higher Carmeli categories, which reflect nosocomial exposure and MDR risk, contain important prognostic information, likely due to delays or inadequacy of initial antimicrobial therapy—a well-known factor contributing to increased mortality in sepsis [16,27,28].

Regarding the microbiological profile, Gram-negative bacilli were predominant in our cohort, with high frequencies of *Escherichia coli*, *Klebsiella pneumoniae*, and *Acinetobacter baumannii*. These findings are consistent with recent reports from European ICUs, where multidrug-resistant Gram-negative pathogens are the leading cause of severe sepsis [29,30].

Recent multicentre studies report MDR prevalence exceeding 40% in sepsis cases, with Gram-negative bacilli such as *Klebsiella pneumoniae*, *Pseudomonas aeruginosa*, and *Acinetobacter baumannii* dominating [8,31]. Mortality remains consistently higher in patients with resistant pathogens, particularly when initial antibiotic coverage is inappropriate [32,33].

Baseline laboratory parameters showed limited prognostic discrimination, supporting previous findings that single admission values often lack predictive.

This finding aligns with multiple studies indicating that baseline values at ICU entry often lack discriminatory power, probably due to the wide biological variability of sepsis at presentation and the influence of pre-hospital interventions [34,35].

For example, white cell blood count (WBC), while easy to obtain and commonly used, is nonspecific and can be elevated due to non-infectious causes as well. A recent review highlights that, although leucocytosis signals inflammation, its sensitivity and specificity for sepsis outcomes are low, and it should be interpreted carefully in a clinical setting [36,37].

Furthermore, corticosteroid therapy can cause significant leucocytosis that peaks around 48 h after administration and may hide the underlying inflammatory trend [38].

Beyond single values, trajectory-based analyses seem more informative: in septic shock, patients with increasing WBC trajectories during the first week had significantly higher mortality, highlighting the importance of monitoring dynamic changes rather than relying solely on admission values [36]. These insights clarify why baseline WBC (and related markers like platelets) in our cohort did not predict outcomes at ICU entry.

During the first 72 h of ICU stay, we observed diverging biomarker trajectories between survivors and non-survivors. The most noticeable pattern was in the NLR: survivors showed a gradual decline, indicating a partial resolution of inflammation, while non-survivors maintained persistently high values. These results are consistent with recent prospective cohorts, which consistently show that NLR values between day 3 and day 5 are better at predicting ICU mortality than baseline measurements [39,40]. In a large multicentre analysis, patients with persistently elevated NLR beyond 72 h had a 2- to 3-fold higher risk of death compared to those with declining values, regardless of traditional severity scores [41]. The biological rationale is strong: elevated and persistent NLR indicates ongoing neutrophilia and sustained lymphopenia, key signs of immune dysregulation in sepsis, which increase the risk of secondary infections and multi-organ failure [42].

Platelet dynamics provide additional prognostic information. Although admission values were similar across groups, non-survivors experienced a significant decline in platelet counts within the first 72 h, while survivors maintained stable levels. This finding supports recent analyses showing that declining platelet trends, rather than baseline counts, predict mortality [10,43]. Moreover, trajectory-clustering studies have shown that patients with persistent thrombocytopenia or steep declines face the highest mortality risk, even after adjusting for SOFA scores [44,45].

When evaluated as prognostic tools at 72 h, NLR clearly outperformed other routine biomarkers, showing very high specificity but only moderate sensitivity. This performance profile suggests that NLR is especially useful for identifying patients at high risk of poor outcomes, although its modest sensitivity limits its effectiveness as a general screening tool. Recent prospective cohorts and meta-analyses, including the comprehensive study by Wu et al. (2024), have indicated that day-3 to day-5 NLR cut-offs typically range between 9 and 15, with higher values consistently linked to increased mortality, supporting the external validity of our findings [46].

In contrast, platelet count and albumin demonstrated limited ability to discriminate when used as single values, which aligns with reports emphasizing their prognostic significance mainly within trajectory-based or composite indices like the platelet-to-lymphocyte ratio (PLR), lactate-to-albumin ratio, or glucose-to-albumin ratio [47,48].

This supports the idea that their predictive contribution should be viewed as complementary, especially when integrated into multivariable risk models rather than interpreted alone. Renal markers (creatinine, eGFR) were significantly linked to outcomes but provided lower AUCs than NLR. This highlights the importance of sepsis-related AKI as a factor in mortality, while also reflecting the known limitations of creatinine-based estimates in critically ill patients, where muscle mass loss and fluid shifts can interfere with renal assessment [49,50].

In multivariable analysis, only the Carmeli score remained an independent predictor of mortality, while SOFA and APACHE II lost significance. This highlights that traditional severity indices, although strongly linked to outcomes in univariate analysis, may not fully capture the complex risk landscape of sepsis when antimicrobial resistance is common. The Carmeli score, provide prognostic insight as a surrogate for prior healthcare exposure and the likelihood of inadequate empirical coverage rather than a direct measure of multidrug resistance, a mechanism repeatedly associated with increased mortality [8,51]. In this way, Carmeli acts less as a direct severity index and more as an indirect indicator for MDR risk, consistent with its original purpose as a predictor of inappropriate empiric therapy.

Recent reviews emphasize that sepsis involves both hyperinflammation and early immunosuppression, highlighting the importance of immune stratification for therapeutic decision-making [52,53].

The integrated model combining APACHE II at admission with the 72-h NLR showed the highest discriminative performance in our cohort, surpassing the predictive accuracy of each parameter alone. However, this combined model should be interpreted with caution, as it was developed from a relatively small single-center cohort without external validation and should therefore be considered exploratory. After bootstrapping by 1000 samples, the NLR at 72 h proved not to be a good discriminative marker in our study. Additionally, power analysis showed that detecting such a small effect (OR = 1.113 for APACHE II and OR = 1.053 for NLR) would require a larger sample size, prompting further studies to include a higher number of patients. Including a greater number of patients in future studies may further validate or challenge our model and its parameters, while also enhancing its discriminative capabilities power. This model emphasizes the complementary roles of clinical severity indices and dynamic immune-inflammatory markers: while APACHE II indicates baseline organ dysfunction, NLR reflects the immune dysregulation trajectory during the early phase of sepsis. Recent prospective studies support this synergistic approach, reporting that hybrid models (clinical score + immune biomarker) achieve significantly higher AUCs and better calibration compared to individual parameters [54,55]. In this context, our findings support the integration of simple immune-inflammatory markers such as NLR and platelet dynamics with MDR risk stratification through the Carmeli score.

The rationale is biologically plausible: sepsis prognosis depends not only on the extent of organ dysfunction but also on the persistence of immune imbalance, which NLR dynamics quantify more effectively than static measures. Clinically, this implies that prospective patients presenting with both a high APACHE II score and persistently elevated NLR at 72 h represent an extremely high-risk subgroup, warranting intensified monitoring, early escalation of supportive therapy, and consideration of adjunctive or experimental interventions. Conversely, a declining NLR despite a high admission APACHE II may identify patients with greater recovery potential.

The absence of systematic MDR phenotyping limits the interpretation of the Carmeli score as a direct predictor of infection or resistance, supporting its role as a surrogate marker of prior healthcare exposure and MDR risk. Although microbiological data were collected for most patients, detailed analysis of resistance phenotypes or pathogen-specific mortality associations was not feasible due to incomplete records in this retrospective cohort. These data were used mainly to confirm the infection source rather than to characterize antimicrobial resistance patterns.

Although combining the Carmeli score with APACHE II or NLR improved mortality prediction, these models should be considered exploratory and need external validation in larger multicenter cohorts before they are used routinely. If prospectively confirmed, such integrated tools could improve early sepsis triage by merging antimicrobial risk assessment with immune-inflammatory dynamics, aiding timely treatment decisions and risk stratification, especially in resource-limited ICU settings. Because this study is from a single center and retrospective, the proposed integrated model should also be seen as preliminary. External validation in larger, multicenter prospective cohorts is crucial to verify their predictive accuracy and applicability before clinical use.

### 4.1. Limitations

#### 4.1.1. Retrospective Nature and Design Limitations

This study has several limitations. It was a retrospective, single-center analysis with a modest sample size, which limits generalizability and increases the risk of model overfitting. To assess biomarker trajectories, only patients with complete data at 0, 48, and 72 h were included; this may have introduced selection bias, as patients with very short ICU stays were underrepresented. Continuous or daily biomarker monitoring could offer more detailed insights into sepsis kinetics, but this was not feasible in the current retrospective design. The 72-h interval was chosen because it aligns with standard reassessment points in sepsis management protocols, reflecting early therapy response and enabling dynamic evaluation of inflammatory and hematological changes.

#### 4.1.2. Sample Size and Unicentric Focus

Although the sample size allowed exploratory modeling, the absence of internal validation and the potential for overfitting remain limitations that require confirmation in larger multicenter cohorts.

#### 4.1.3. Lack of Treatment Oversight

Detailed therapeutic variables, such as timing of antibiotics, vasopressor or corticosteroid use, and mechanical ventilation, were not available, preventing adjustment for potential confounders. These unmeasured therapeutic factors are known to strongly influence sepsis outcomes and could have introduced residual confounding, which limits the ability to fully assess the independent prognostic value of the studied biomarkers. Future prospective multicenter studies should include these variables to improve model robustness and clinical applicability.

#### 4.1.4. Lack of External Validation

Finally, the models were not externally validated, so the results should be interpreted as exploratory and hypothesis-generating. Another limitation is the lack of advanced immunological profiling, which could have provided a more detailed characterization of host immune dysregulation in sepsis. Additionally, the lack of systematic MDR phenotyping limits the ability to interpret the Carmeli score as a direct predictor of infection or resistance, emphasizing its role as a surrogate marker for previous healthcare exposure and MDR risk.

### 4.2. Clinical Implications and Future Directions

Despite its exploratory nature, this study emphasizes the potential usefulness of combining severity scores with dynamic biomarker trajectories for early risk assessment in sepsis. The independent predictive link of the Carmeli score with mortality indicates that indices reflecting MDR risk, traditionally used to guide empiric therapy, may also have prognostic value. Incorporating this aspect with established clinical indices and dynamic inflammatory markers could improve early risk stratification in sepsis. Beyond hematologic indices such as NLR, emerging biomarkers, including selected microRNAs—such as miR-146a, miR-150, miR-223, and miR-4722–5p—are connected to immune dysregulation and sepsis outcomes [56,57]. Integrating MDR risk scores with immune-inflammatory markers may not only improve prognostic accuracy but also support therapeutic decision-making, particularly in guiding timely empirical antimicrobial therapy and identifying candidates for adjunctive immunomodulatory approaches.

Future multicentre prospective studies are necessary to validate these findings, determine optimal biomarker thresholds, and assess their incorporation into a risk-based clinical decision framework.

## 5. Conclusions

Combining the Carmeli score with dynamic immune-inflammatory markers, such as NLR and platelet trajectories, improves early prognostic stratification in sepsis. This integrated method may support more accurate risk assessment and guide timely empirical treatment, especially in patients at risk for multidrug-resistant infections. Validation in larger multicenter prospective studies is needed before clinical use.

## Figures and Tables

**Table 1:** Baseline characteristics of the study population.

Characteristic	Total (*n* = 120)	Survivor (*n* = 50)	Non-Survivor (*n* = 70)	*p*-Value
Age (years, median [IQR])	70.5 (62–81)	67 (52.5–79.75)	72 (64–81)	0.144
Male sex, *n* (%)	63 (52.5)	26 (52)	37 (52.8)	0.926
SOFA score (mean ± SD)	13.13 ± 3.91	11 ± 3.71	14.61 ± 3.34	<0.001
APACHE II score (median [IQR])	27 (22–35)	22 (18–28)	30.5 (25.75–38)	<0.001
Carmeli score (median [IQR])	2 (2–3)	2 (2–3)	2 (2–3)	0.001 †
ICU length of stay (days)	6 (4−12.75)	6.5 (4–13.25)	6 (4–11.25)	0.483
Cardiovascular comorbidity, *n* (%)	102 (85)	42 (84)	60 (85.71)	0.795
Pulmonary comorbidity, *n* (%)	76 (63.33)	34 (68)	42 (60)	0.370
Renal comorbidity, *n* (%)	46 (38.33)	17 (34)	29 (41.43)	0.409
Neurological comorbidity, *n* (%)	48 (40)	19 (38)	29 (41.43)	0.705
Digestive comorbidity, *n* (%)	46 (38.33)	17 (34)	29 (41.43)	0.409
Hematologic comorbidity, *n* (%)	46 (38.33)	20 (40)	26 (37.14)	0.751
Diabetes mellitus, *n* (%)	35 (29.17)	14 (28)	21 (30)	0.812
Urologic comorbidity, *n* (%)	35 (29.2)	13 (26)	22 (31.4)	0.519
Oncologic comorbidity, *n* (%)	16 (13.33)	5 (10)	11 (15.71)	0.424
Psychiatric comorbidity, *n* (%)	8 (6.67)	4 (8)	4 (5.71)	0.718
COVID-19 history, *n* (%)	5 (4.17)	1 (2)	4 (5.71)	0.400

Abbreviations: SOFA—Sequential Organ Failure Assessment; Acute Physiology and Chronic Health Evaluation II; ICU—intensive care unit; *n*—number of patients; IQR—interquartile range. † Mann–Whitney U test comparing Carmeli scores between survivor and non-survivor groups yielded a *p*-value of 0.001. Although median scores were identical (2 IQR 2–3), the test compared rank distributions and found a significant difference, indicating that non-survivors tended to have higher Carmeli scores overall despite similar medians.

**Table 2:** Laboratory parameters at admission (T0).

Parameter Median (IQR)	Total (*n* = 120)	Survivor (*n* = 50)	Non-Survivor (*n* = 70)	*p*-Value
WBC (×10^9^/L)	12.72 (8.4–19.3)	12.75 (7.67–20.17)	12.72 (8.66–18.5)	0.977
Neutrophils (×10^9^/L)	10.87 (7.02–16.31)	10.38 (5.53–16.78)	10.95 (7.17–15.84)	0.598
Lymphocyte (×10^9^/L)	0.85 (0.42–1.25)	0.93 (0.55–1.39)	0.76 (0.4–1.21)	0.147
NLR	12.74 (7.23–25.22)	10.71 (7.04–20.96)	17.19 (7.55–31.41)	0.076
PLT (×10^9^/L)	200 (144.5–306)	214.5 (163.25–302.75)	198 (138–307.5)	0.6
Hemoglobin (g/dL)	10.95 (8.3–12.75)	11.2 (8.25–13.33)	10.85 (8.25–12.33)	0.375
Albumin (g/L)	24 (21.18–30.93)	26 (22.3–31.5)	23.3 (19.8–30.2)	0.127
Creatinine (mg/dL)	1.25 (0.79–2.19)	0.98 (0.72–2.11)	1.34 (0.91–2.62)	0.241

Abbreviations: WBC—white blood cell count; NLR—neutrophil-to-lymphocyte ratio; PLT—platelet count.

**Table 3:** 72-h dynamics of hematologic and inflammatory markers.

Parameter (Median [IQR])	T0 (Admission)	T1 (48h)	T2 (72 h)	*p*-Trend (Friedman/Post Hoc))
PLT Survivors (×10^9^/L)	216 (179–338.1)	213 (166.5–291)	200 (146.5–306)	0.572
PLT– non-survivors (×10^9^/L)	198 (145.25–307.5)	209 (146.75–321.25)	195 (85.5–262.25)	0.001 (T0–T1 = 0.038; T1–T2 < 0.001, T0–T2 < 0.001)
NLR Survivors	10.71 (7.04–20.96)	9.3 (5.92–17.4)	6.92 (4.75–12.08)	<0.001 (T0–T1 = 0.197; T1–T2 < 0.001; T0–T2 < 0.001)
NLR–non-survivors	17.19 (7.55–31.41)	15.97 (7.35–28.13)	18.89 (8.73–31.01)	0.929

Abbreviations: NLR—neutrophil-to-lymphocyte ratio; PLT—platelet count; h—hours. All longitudinal comparisons were performed using the Friedman test (non-parametric). When the overall *p* < 0.005, post hoc pairwise contrasts were conducted with the Wilcoxon signed-rank test. Values are reported as median [IQR].

**Table 4:** ROC curve analysis of biomarkers at 72 h for the prediction of mortality.

Parameter (72 h)	AUC	95% CI	*p*-Value	Sensitivity (%)	Specificity (%)	Cut-Off
NLR	0.765	0.668–0.863	<0.001	53.7	92.5	17.07
PLT	0.566	0.450–0.682	0.273	27.8	95.1	99
Albumin	0.634	0.487–0.782	0.084	59.4	80	23.9
Creatinine	0.649	0.532–0.766	0.014	73.1	63.4	0.755
eGFR	0.637	0.521–0.753	0.024	88.5	73.2	93.8

Abbreviations: AUC—area under the curve; CI—confidence interval; PLT—platelet count; NLR—neutrophil-to-lymphocyte; eGFR—estimated glomerular filtration rate; Units—PLT (×10^9^/L); Albumin (g/L); Creatinine (mg/dL); (eGFR) (mL/min/1.73 m^2^). Cut-offs were determined using the Youden index. AUC values are shown with 95% CI.

**Table 5:** Univariate logistic regression analysis of clinical severity scores for mortality prediction.

Predictor	B	S.E.	Wald	*p*-Value	OR (Exp(B))	95% CI for OR	BCa 95% CI
Carmeli Score	1.304	0.429	9.243	0.002	3.68	1.60–8.47	1.6–11.15
SOFA Score	0.130	0.106	1.522	0.217	1.14	0.92–1.42	0.8–1.41
APACHE II Score	0.082	0.043	3.643	0.056	1.09	0.99–1.19	1–1.35
Constant	−6.828	1.495	20.846	<0.001	0.001	–	–

B—regression coefficient; S.E.—standard error; Wald—Wald chi-square statistic; OR—odds ratio (exponentiated coefficient); 95% CI—conventional 95% confidence interval for OR; BCa 95% CI—bias-corrected and accelerated 95% CI calculated from 1000 resampled datasets for OR. Confidence intervals that exclude 1 indicate statistical significance at the 0.05 level. *p*-values correspond to two-tailed tests. The constant term represents the model intercept. Dependent variable—in-hospital mortality (1 = death). OR are for a 1-point increase in each score.

**Table 6:** Logistic regression combining APACHE II and NLR at 72 h for predicting mortality.

Predictor	B	S.E.	Wald	*p*-Value	Adjusted OR	95% CI for OR	BCa 95% CI
APACHE II Score	0.107	0.034	10.075	0.002	1.113	1.04–1.20	1.02–1.26
NLR at 72 h	0.052	0.024	4.653	0.031	1.053	1.00–1.10	0.049–1.2
Constant	−3.363	0.922	13.310	<0.001	0.035	–	–

NLR—neutrophil-to-lymphocyte ratio; B—regression coefficient; S.E.—standard error; Wald—Wald chi-square statistic; OR—odds ratio (exponentiated coefficient); 95% CI—conventional 95% confidence interval for OR; BCa 95% CI—bias-corrected and accelerated 95% CI calculated from 1000 resampled datasets for OR. Confidence intervals that exclude 1 indicate statistical significance at the 0.05 level. *p*-values correspond to two-tailed tests. The constant term represents the model intercept. Dependent variable—in-hospital mortality (1 = death). OR are for a 1-point increase in each score. Adjusted ORs per 1-point increase in APACHE II and per 1-unit increase in NLR-72 h.

## Data Availability

Data are available upon request from the corresponding author.

## Supplementary Materials

The following supporting information can be downloaded at: https://www.mdpi.com/article/10.3390/biomedicines13112658/s1. Figure S1, ROC curve of platelet count (PLT) at 72 h for prediction of mortality; Figure S2, ROC curve of albumin at 72 h for prediction of mortality; Table S1, Diagnostics for the multivariable scores model (Carmeli, Sofa, APACHE II) predicting in-hospital mortality; Table S2, Multivariable logistic regression model predicting in-hospital mortality using Carmeli, SOFA, and APACHE II scores, adjusted for age, sex, and comorbidities; Table S3, Diagnostics for the combined logistic regression model (APACHE II + NLR at 72 h) predicting in-hospital mortality; Table S4, Multivariable logistic regression model predicting in-hospital mortality using APACHE II scores and NLR at 72 h, adjusted for age, sex, and comorbidities; Table S5, Distribution of types of infections in the study group; Table S6, Distribution of types of infections in the study group, assessed at 72 h after ICU admission. Percentages are expressed relative to the total study cohort (*n* = 120).

## Abbreviations

The following abbreviations are used in this manuscript:

Sepsis-3	Third International Consensus Definitions for Sepsis and Septic Shock
ICU	Intensive Care Unit
SOFA	Sequential Organ Failure Assessment (score)
APACHE II	Acute Physiology and Chronic Health Evaluation II (score)
CRP	C-reactive protein
PCT	Procalcitonin
WBC	White blood cell count
PLT	Platelet count
NLR	Neutrophil-to-Lymphocyte Ratio
PLR	Platelet-to-Lymphocyte Ratio
INR	International Normalized Ratio
eGFR	Estimated Glomerular Filtration Rate
MDR	Multidrug-Resistant (organisms)
MSSA	Methicillin-Susceptible Staphylococcus aureus
MRSA	Methicillin-Resistant Staphylococcus aureus
spp.	Species (plural; used for a genus with multiple species)
TNF-α	Tumor Necrosis Factor alpha
IL-6	Interleukin-6
ROC	Receiver Operating Characteristic (curve)
AUC	Area Under the Curve
OR	Odds Ratio
CI	Confidence Interval
SD	Standard Deviation
IQR	Interquartile Range
ANOVA	Analysis of Variance
AKI	Acute Kidney Injury
SPSS	Statistical Package for the Social Sciences
B	Regression coefficient (in logistic regression)
S.E.	Standard Error
T0/T1/T2	Timepoints: admission (T0), 48 h (T1), 72 h (T2)
