# Supplementary material for: Early Diagnostic Markers and Risk Stratification in Sepsis: Prognostic Value of Neutrophil-to-Lymphocyte Ratio, Platelets, and the Carmeli Score
Source: Biomedicines. 2025 Oct 29;13(11):2658. doi: 10.3390/biomedicines13112658 (PMC12649956; doi:10.3390/biomedicines13112658)
Supplement: Supplementary file 1 [file biomedicines-13-02658-s001.zip › biomedicines-3908336-supplementary.pdf]

biomedicines

MDPI

Supplementary Material

Biological Predictors of Mortality in Sepsis: The Prognostic Value of Dynamic Hematological and Inflammatory Markers in Intensive Care

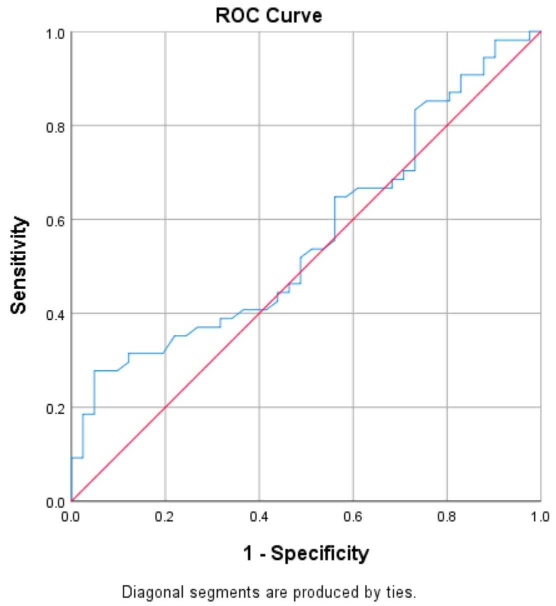

Supplementary Figure S1. ROC curve of platelet count (PLT) at 72 h for prediction of mortality. PLT did not reach statistical significance.

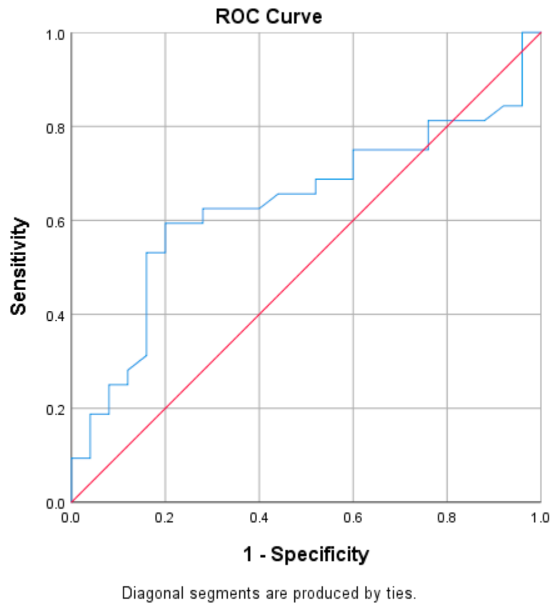

Supplementary Figure S2. ROC curve of albumin at 72 h for prediction of mortality. Albumin showed borderline, non-significant predictive performance.

 https://doi.org/10.3390/biomedicines13112658

Supplementary Table S1. Diagnostics for the multivariable scores model (Carmeli, Sofa, APACHE II) predicting in-hospital mortality

Metric	Value
Omnibus test of model coefficients	$\chi^2 = 40.836$, df = 3, p < 0.001
Pseudo-R^2 (Cox& Snell)	0.290
Pseudo-R^2 (Nagelkerke)	0.391
Hosmer-Lemeshow goodness-of-fit	$\chi^2 = 14.688$, df = 8, p = 0.066
ROC AUC	0.821 (SE = 0.040; 95% CI 0.743-0.899; p< 0.001
Classification at thresholod 0.50	
Overall accuracy	75.6%
Sensitivity (death)	80.0%
Specificity (survivor)	69.4%
Youden-derived optimal threshold	0.659
Sensitivity at optimal threshold	71.4%
Specificity at optimal threshold	85.7%
Youden index	0.571
Collinearity	VIF
Carmeli score	1.053
SOFA score	3.32
APACHE II score	3.225

VIF –variance inflation factors

Notes: Metrics refer to multivariable model includes Carmeli, SOFA, and APACHE II as predictors. ROC AUC refers to the model's overall discriminative ability. Optimal probability threshold (Youden) is the cut-off on the model-predicted probability that maximizes Sensitivity + Specificity – 1.

Supplementary Table S2. Multivariable logistic regression model predicting in-hospital mortality using Carmeli, SOFA, and APACHE II scores, adjusted for age, sex, and comorbidities.

Variable	B	S.E.	Wald	p	Exp(B)	95% C.I.for EXP(B)	BCa 95% CI
Carmeli Score	1.267	0.442	8.198	0.004	3.549	1.491 - 8.447	1.471 - 11.989
SOFA Score	0.14	0.108	1.686	0.194	1.15	0.931 - 1.42	0.801 - 1.511
APACHE II Score	0.076	0.044	3.041	0.081	1.079	0.991 - 1.176	1.009 - 1.302
Comorbidities	0.016	0.169	0.009	0.925	1.016	0.729 - 1.416	0.712 - 1.402
Age	0.013	0.017	0.585	0.444	1.013	0.98 - 1.048	0.966 - 1.058
Sex	0.203	0.464	0.191	0.662	1.225	0.493 - 3.043	0.471 - 3.677
Constant	-7.677	1.888	16.532	<.001	0		

Supplementary Table S3 Diagnostics for the combined logistic regression model (APACHE II + NLR at 72 h) predicting in-hospital mortality

Metric	Value
Omnibus test of model coefficients	χ^2 = 29.108, df = 2, p < 0.001
Pseudo-R^2 (Cox&Snell)	0.266
Pseudo-R^2 (Nagelkerke)	0.358
Hosmer-Lemeshow goodness-of-fit	χ^2= 4.410, df = 8 , p = 0.818
ROC AUC	0.827 (95% CI 0.742-0.912; p < 0.001)
Classification at threshold 0.50	
Overall classification accuracy	75.5%
Sensitivity (deaths correctly identified)	79.6%
Specificity (survivors correctly identified)	70.0%
Youden-derivated optimal threshold	0.62
Sensitivity at optimal threshold	68.5%
Specificity at oprimal threshold	87.5%
Youden index	0.571
Collinearity	VIF
APACHE II score	1.125
NLR at 72h	1.126

VIF –variance inflation factors

Predictors: APACHE II (per 1 -point increase) and NLR at 72 h (per 1 – unit increse). ROC AUC refers to the model's overall discriminative ability. Optimal probability threshold and the associated sensitivity/specificity correspond to the cutoff derivated by the Youden index.

Supplementary Table S4. Multivariable logistic regression model predicting in-hospital mortality using APACHE II scores and NLR at 72h, adjusted for age, sex, and comorbidities.

Variable	B	S.E.	Wald	Sig.	Exp(B)	95% C.I.for EXP(B)	BCa 95% CI
NLR at 72h	0.051	0.024	4.4	0.036	1.053	1.003 - 1.104	0.999 - 1.192
APACHE II Score	0.11	0.036	9.262	0.002	1.116	1.04 - 1.197	1.039 - 1.259
Comorbidities	-0.104	0.199	0.274	0.601	0.901	0.61 - 1.331	0.551 - 1.409
Age	0.013	0.018	0.483	0.487	1.013	0.977 - 1.049	0.976 - 1.058
Sex	0.065	0.499	0.017	0.896	1.068	0.401 - 2.84	0.384 - 3.924
Constant	-3.879	1.469	6.97	0.008	0.021		

Supplementary Table S5. Distribution of types of infections in the study group

Infection type	N (%)	Survivors (n)	Deceased (n)	Mortality (%)
Germ not identified at 72 h	34 (25.8)	15	16	51.6
Gram - positive	17 (14.2)	9	8	47.0
Gram - negatives	57 (47.5)	19	38	66.7
Mixed (G+/G-)	15 (12.5)	7	8	53.3
Total	120 (100)	50	70	58.3

Supplementary Table S6. Distribution of types of infections in the study group, assessed at 72 h after ICU admission. Percentages are expressed relative to the total study cohort (n = 120)

Agent pathogen	N (%)
E.coli	16 (13.3)
Klebsiella pneumoniae	16 (13.3)
Acinetobacter baumanni	13 (10.8)
Pseudomonas aeruginosa	11 (9.2)
Staphylococus aureus MSSA	20 (16.7)
MRSA	4 (3.3)
Enterococcus spp.	10 (8.3)
Streptococcus pneumoniae	5 (4.2)
Clostridioides difficile	14 (11.7)
Candida spp.	32 (26.7)
Other rare bacteria (<2% each)	15 (12.5)
